# Evaluation of Patched-1 Protein Expression Level in Low Risk and High Risk Basal Cell Carcinoma Subtypes

**DOI:** 10.31557/APJCP.2019.20.9.2851

**Published:** 2019

**Authors:** Rowida Almomani, Mariam Khanfar, Khaldon Bodoor, Firas Al-Qarqaz, Mohammad Alqudah, Hanan Hammouri, Asma Abu-Salah, Yazan Haddad, Wisam Al Gargaz, Ziyad M Mohaidat

**Affiliations:** 1 *Department of Medical Laboratory Sciences,*; 2 *Department of Applied Biology,*; 3 *Department of Dermatology,*; 4 *Department of Pathology, *; 5 *Department of Mathematics and Statistics, Faculty of Science and Arts,*; 6 *Orthopedic Surgery Division, Special Surgery Department, Faculty of Medicine, Jordan University of Science and Technology, Irbid, Jordan. *

**Keywords:** Basal cell carcinoma, PTCH1, high risk BCC, low risk BCC

## Abstract

**Objective:**

Basal cell carcinoma (BCC) is the most common malignancy in humans and represents a growing public health care problem. The major etiological factors contributing to BCC development are exposure to ultraviolet radiation and genetic alterations. BCC is primarily caused by dysregulation of sonic Hedgehog (HH) signaling pathway in basal cells of the skin. BCC can be classified into low risk non-aggressive and high risk aggressive subtypes. BCC subtypes differentiation is essential for prognosis and for better disease management and treatment strategies. The aim of this study was to assess the correlation between PTCH1 protein expression level and the aggressiveness of BCC histopathology.

**Methods:**

Archival paraffin embedded blocks containing BCC were retrieved from a cohort of 101 patients. Immunohistochemistry staining was performed to assess the expression level of PTCH1 which is a key component of Hedgehog pathway.

**Results:**

101 paraffin embedded samples were evaluated and classified as high risk and low risk BCC subtypes by histopathological finding. High risk BCC subtypes were found in 40 samples (39.6%) and low risk subtypes were identified in 61 samples (60.4%). Nodular was the most frequent subtype which was found in (56/ 101), followed by infiltrative (22/101) and micronodular (14/ 101) subtypes. Positive PTCH1 expression was found highest in nodular subtypes (46.5%).

**Conclusion:**

In this study, the correlation between low risk or high risk BCC subtypes and PTCH1 expression level was not statistically significant (p>0.05), but the frequency of positive PTCH1 expression was found to be higher in low risk subtypes than high risk BCC subtypes.

## Introduction

Basal Cell Carcinoma (BCC) is the most common non-melanoma skin cancer and it is the most common skin cancer worldwide. BCC arises from basal cells present in the lower layer of the epidermis. In fair-skinned populations, BCC is by far the most prevalent skin cancer making around 80% of these tumors. Despite being the most common skin cancer, luckily it has almost no potential for metastasis and hence it is rarely fatal (Al-Qarqaz, 2019; Böni et al., 2002; Rubin et al., 2005). However, this malignancy causes considerable morbidity especially when the tumor is located near important sites and poses a huge burden on healthcare systems in many parts of the world (Al-Qarqaz et al., 2018; Rubin et al., 2005).

The incidence of BCC is still rising with almost 10% each year around the world, for example in the Netherlands the incidence increased by three folds between 1973 and 2008 from 40 to 148 per 100,000 in males and from 34 to 141 in females (Flohil et al., 2011). The major etiological factors contributing to BCC development are the exposure to ultraviolet radiation and genetic alterations. BCC lesions usually occur in sun-exposed areas of the body such as the head and neck regions. Beside ultraviolet radiation exposure and genetic predisposition, there are other recognized risk factors associated with BCC which include immunosuppression, male sex, increasing age, fair skin color, and arsenic exposure (Boukamp, 2005; D’Orazio et al., 2013; Dourmishev et al., 2013; Gailani et al., 1996; Raasch et al., 2006; Skoda et al., 2018; Zhang et al., 2001).

Several key genes have been implicated in the molecular pathogenesis of BCC, especially proto-oncogenes and tumor suppressor genes. These include* PTCH1* and *SMO* genes which are key components of the Hedgehog (Hh) pathway, *TP53* tumor suppressor gene, and other RAS proto-oncogenes family members. Hh pathway has a significant role during embryogenesis; it plays a major role in controlling several normal cellular processes, comprising cell proliferation, dif-ferentiation, organogenesis and body patterning. In addition, Hh signaling pathway is involved in tissue repair, regeneration and stem cell maintenance in adult tissues (Aszterbaum et al., 1998; Johnson et al., 1996; Lacour, 2003; Noubissi et al., 2018)

The first link between Hh signaling imbalances and cancer stemmed from studies of patients with a hereditary syndrome known as Nevoid basal cell carcinoma syndrome (NBCCS) or Gorlin syndrome. NBCCS is a rare autosomal dominant hereditary syndrome that characterized by predisposition to develop specific types of cancer such as multiple BCC in young age and medulloblastoma. Several studies showed that loss of function mutations in the *PTCH1* gene can lead to BCC predisposition in the NBCCS (Bresler et al., 2016; Dong et al., 2000; Fujii et al., 2003; Hahn et al., 1996; Wolter et al., 1997). *PTCH1 *gene is located on chromosome 9q22.3, contains 23 exons that spans about 74 kb. It encodes for a transmembrane protein composed from 1,447 amino acids which acts as a key receptor for Hh signaling pathway (Gailani et al., 1992). A number of studies have also shown that sporadic BCCs have a high frequency of loss-of-function mutations and loss of heterozygosity (LOH) in *PTCH1* gene which is due to copy loss or uniparental disomy. These studies support the notion that PTCH1 functions as a tumor suppressor gene (Adolphe et al., 2006).

Histopathologically, BCCs can be grouped into different subtypes based on the different growth patterns which include nodular, superficial, morphoeic, micronodular, infiltrative and basosquamous forms. These histopathological subtypes can be low risk, for instance nodular and superficial to high risk forms such as infiltrative, micronodular, basosquamous, and morpheaform which are mainly associated with aggressive tissue invasion. BCC subtypes differentiation is essential for prognosis and for better disease management and treatment strategies (Al-Qarqaz et al., 2018; Marcelina, 2016; Raasch et al., 2006; Walling et al., 2004). 

In this study, we aimed to assess the level of expression of PTCH1 protein in high risk and low risk BCC subtypes. Paraffin-embedded tissues from BCC patients were collected and used to evaluate the expression levels of PTCH1 protein by immunohistochemistry analysis.

## Materials and Methods

This study was performed on archival paraffin embedded blocks of 101 samples of BCC from the department of pathology in King Abdullah University Hospital, Jordan University of Science and Technology, Jordan, between the years 2010 and 2017. All included cases were diagnosed as BCC by certified pathologists. Complete pathological and clinical data were obtained for all patients from King Abdullah University Hospital and were available for further analysis. 

Paraffin embedded tissues from patients diagnosed with BCC were collected and used in this study for immunohistochemistry (IHC) for PTCH1 protein expression analysis. The tissue samples were cut into 4-μm thick sections on coated slides using Accu-Cut^® ^SRM^™^ 200 rotary microtome and prepared for IHC. Sample sections were de-paraffinized in xylene twice for 30 minutes, then rehydrated in a series of graded alcohol from a concentration of 100% into 70% for three minutes each, followed by two minutes incubation in a distilled water tank.

Antigen retrieval took place in pressure cooker at 122.5°C. Slides were then treated with 3% hydrogen peroxide for 15 minutes for inactivation of endogenous peroxidase then rinsed with PBS for 5 minutes. Rabbit polyclonal to Patched-1 (Ab53715: Abcam, UK) was then added for one hour (Dilution 1:200), rinsed with PBS. For the secondary antibody, poly HRP conjugate from Sakura was used for 30 minutes and then slides were rinsed with PBS. Immunoreactivity was visualized with 3,3diaminobenzidine (DAB) chromagen and then counterstained with

Mayer’shematoxylin. Finally, the sample sections were dehydrated, then mounted with diamount solution. In all specimens, ten fields were examined at ×400 magnification and at least 100 cells were evaluated in each area in order to assess the tumor as a whole. Immuno-reactivity evaluation was carried out in a similar manner to Marcelina et al. (Marcelina, 2016). In brief, area percentage of stained cells was scored as: 0: no expression (no staining), 1: positive staining in 1-25% of the cells, 2: positive staining in 26-50% of the cells, 3: positive staining in 51-75% of the cells, and 4: positive staining in 76-100% of the cells. The staining intensity was scored as: 1 indicated weak, 2 indicated moderate, and 3 denoted strong. The final scoring of PTCH1 protein expression level was obtained by the multiplication of area percentage with staining intensity score and categorized as: Negative/weak (0-4), Positive (moderate/ Strong (5-12)).

Histology and Immuno-stained sections were comprehensively reviewed and examined under the light microscope by two independent certified pathologists.

*Statistical analysis*

In order to assess the correlation between histopathological features and PTCH1 expression level, statistical analysis was done. The JMP Statistics program (version 14) and SAS (version 9.4) were utilized doing the analyses. Statistical summaries were found for all variables. Shapiro-Wilk W test was used to assessing normality for the only continuous variable age since normality is a crucial assumption to use ANOVA and t-test. Levene test was used to check equal variances assumption to use ANOVA. Evaluation of the relationship between age, and PTCH1 expression, high or low risk (aggressiveness) or sex was performed via t-test. The relationship between age and BCC subtypes was evaluated via ANOVA test. Furthermore, we used the Chi-square and the Fisher exact tests of independence to assess the association between nominal variables. Logistic regression aside to odds ratio was used to study the association for age and PTCH1 expression, high or low risk (aggressiveness).

## Results

A total of 101 BCC cases were included in this study and selected in a retrospective analysis of their pathological records carried out between the years 2010 and 2017 in the Department of Pathology at King Abdullah University Hospital. The cohort included 63 (62%) males and 38 (38%) female cases with a male: female ratio of 1.66:1. The age mean was 62.5±14. The majority of samples (63.4%) have an age distribution of ≥ 60 years. Moreover, the normality for the age variable was tested, and it can be assumed normal (Shapiro-Wilk W P-value =0.1809). 

**Table 1:** Counts and Percentages of the PTCH1 Expression Level in BCC Subtypes

	Count	PTCH1 expression		
	Total %	Negative	Positive	Total
histopathologic subtypes	Basosquamous	0	2	2
		0	1.98	1.98
		0	2.33	
		0	100	
	Fibroepithelioma of Pinkus	0	2	2
		0	1.98	1.98
	Infiltrative	3	19	22
		2.97	18.8	21.78
	Micronodular	3	11	14
		2.97	10.89	13.86
	Morpheaform	0	2	2
		0	1.9	1.9
	Nodular	9	47	56
		8.91	46.53	55.45
	Superficial	0	3	3
		0	2.97	2.97
	Total	15	86	101
		14.85	85.15	
			

**Table 2:** Counts and Percentages of the PTCH1 Expression in High Risk and Low-Risk Subtypes of BCC

		PTCH1 expression		
	Count Total %	Negative	Positive	Total
subtypes	low	9.00	52.00	61
		8.91	51.49	
	high	6.00	34.00	40
		5.94	33.66	
	Total	15.00	86.00	101
		14.85	85.15	

Contingency Table, risk subtypes By Final Score

**Table 3:** Odds Ratio Using Chi-Square Test for Sex and PTCH1 Expression Over Aggressiveness of BCC

Characteristics	Group	Odds ratio	95% confidence interval.	p-value
Sex	Female	1		
	Male	0.846	0.372-1.922	0.69
PTCH1 expression	Negative	1		
	Positive	0.981	0.320-3.005	0.973

**Table 4:** Parameter Estimates and Odds Ratio for Logistic Models

Dependent Variable	Term	Estimate	Unit Odds Ratio	
PTCH1 expression	Intercept	2.181	.	
	AGE	-0.0069	0.993	For log odds of Positive/Negative
aggressiveness of BCC	Intercept	0.94	.	
	AGE	-0.0082	0.992	For log odds of low/high

**Figure 1:**
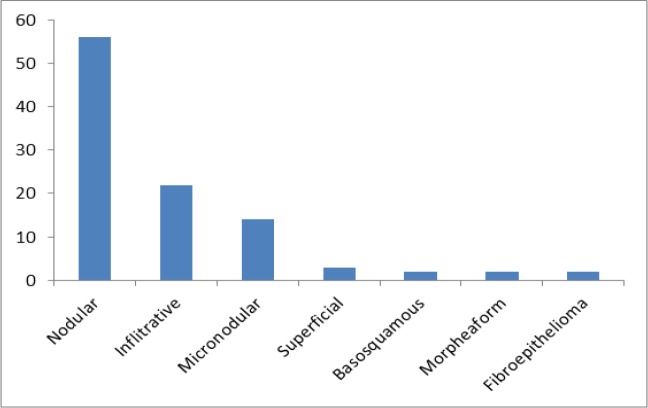
Distribution of Histopathological Subtypes of BCC

**Figure 2:**
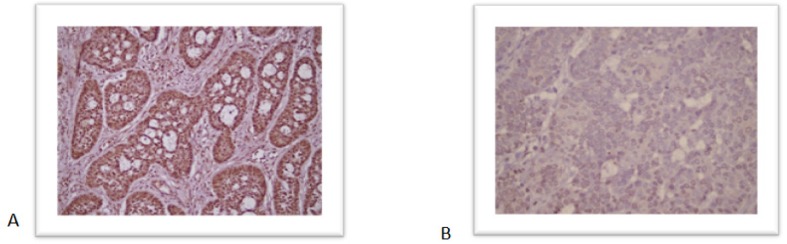
Immunohistochemistry Staining Results of PTCH1 Expression. A, shows strong PTCH1 expression while B shows a negative staining of *PTCH1*

**Figure 3.**
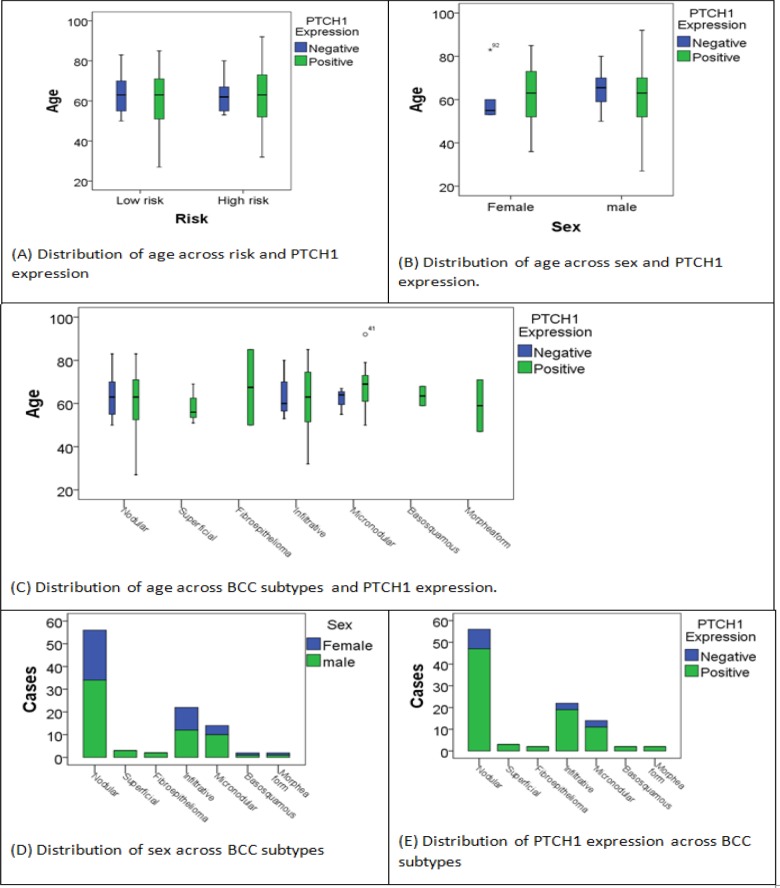
Distribution of PTCH1 Expression Age, Sex, Across Several Variables

In this study, the following histopathologic subtypes were characterized; nodular pattern predominated, accounting for 56 (55.5%) cases, followed by Infiltrative (20.8%) and Micronodular 14 (13.9%). Using a Fisher exact test, a significant association was found between histopathologic subtypes and risk (P-value <0.0001). Records showed that high-risk subtypes BCC was found in 39.6% (40/ 101) samples and low risk subtypes BCC was identified in 60.40% (61/ 101) of samples. A complete analysis of the histopathologic subtypes of BCC is shown in Figure 1. Furthermore, Fibroepithelioma of Pinkus, Superficial, and Nodular are associated with low risk and the rest types with high risk.

IHC staining was undertaken to evaluate the expression of PTCH1 in the tumor tissue and their adjacent histologically normal epithelial cells. The staining intensity was assessed by a pathologist, and a scoring system of negative and positive expression was used in this study. The expression of PTCH1was localized to the membrane, cytosol/ nucleus (Figure 2).

Compared to the adjacent normal epithelium, 86 (85.2%) of the investigated BCC cases exhibited positive PTCH1 protein expression. Fifteen (14.8%) cases showed negative expression (Table 1). 

The percentage of strong PTCH1 expression was found highest in nodular (low risk) BCC subtype (46.5%) followed by Infiltrative and Micronodular (high risk) subtypes, 18.8% and 10.8% respectively (Table 1). Fisher exact test showed insignificant P-value that equals 0.9716. 

We next evaluated the level of PTCH1 expression in Low risk and high risk BCC subtypes. Our data show that the frequency of positive PTCH1 expression was higher in low-risk BCC than high risk BCC subtypes, accounting for 52/101 cases (51.4%) (Table 2). Unfortunately, the chi-square test was insignificant (P-value =0.9729) 

The statistical analysis data showed no significant difference between PTCH1 positive and negative expression groups in terms of mean age (P-value=0.377). According to risk (aggressiveness), no significant difference between high risk and low-risk groups was found in terms of mean age (P-value =0.697). Then for sex, no significant difference between male and female groups age means was found (P-value =0.574). All done using t-test since age is normally distributed. Then for BCC subtypes ANOVA was used since age is normally distributed and there is no evidence for unequal variances between the groups (Levene P-value=0.47). No significant results were found F(8,92)=0.59 with P-value=0.79. That indicates there is no significant difference between groups age means.

Same tests were done but within the risk groups separately. First for high risk group, no significant difference was found between PTCH1 positive and negative expression groups in terms of mean age (P-value=0.512), sex (P-value =0.868), or BCC subtype (Levene P-value=0.105, F(5,34)=0.69 with P-value=0.64). 

Within the low risk groups, no significant difference was found between PTCH1 positive and negative expression groups in terms of mean age (P-value=0.33), sex (P-value =0.23), or BCC subtype (Levene P-value=0.26, F(2,58)=0.29 with P-value=0.75). Distribution of PTCH1 expression age, sex, across several variables, is shown in Figure 3.

The role of clinical characteristics in the aggressiveness of BCC was evaluated by odds ratios using chi-square tests (Table 3). The odds ratio values for sex and PTCH1 expression were close to one-fold in comparison to reference (P-values are 0.690, and 0.973, respectively). 

Lastly, logistic regression models were used along with the odds ratios to check if age can predict *PTCH1* expression and risk. Unfortunately, both are insignificant (P-values are 0.75 and 0.60, respectively). Table 4 contains age estimates along with the odds ratio for both models.

## Discussion

Hedgehog (Hh) signaling pathway has a significant role during embryogenesis; it plays a major role in controlling several normal cellular processes (Oro and Higgins, 2003). Dysregulation of this pathway has been implicated in the etiology of a variety of cancers, including BCC (Barnes et al., 2005; Bonilla et al., 2016; Lacour, 2003). The canonical Hh pathway comprises a number of key elements, including the Hh ligands (Sonic Hh [SHh]), Indian Hh (IHh), Desert Hh, Patched receptor proteins( PTCH1 and PTCH2), the G-protein-coupled-like receptor (smoothened) (SMO), and the GLI1, GLI2,and GLI3 transcriptional factors (Skoda et al., 2018). 

*PTCH1* gene encodes for Patched1 which is a transmembrane protein that functions as a receptor of the secreted signaling molecule SHh and as a negative regulator of Hh signal transduction (Gailani et al., 1992). In the absence of SHh, SMO activity is blocked by direct interaction with Patched. So under inactive signaling conditions, the membrane bound protein PTCH1 maintains SMO in its inactive and dephosphorylated form, leaving it susceptible to endocytosis and degradation. Consequently, preventing the activation of glioma-associated oncogene (GLI) transcription factors (GLI1, GLI2, GLI3). When Hh pathway is activated, SMO is released upon binding of SHH to patched1. This can lead to the initiation of signal transduction cascade that causes activation of the transcription factor Gli1 and allows GLi1 to localize in the nucleus. High level of PTCH1 is linked to elevated Hh pathway activation, thus, dysregulation and constitutive activity of this pathway by inactivating mutations in PTCH1 leads to unrestricted SMO activity and high levels of the Gli1 transcriptional factor, consequently, induces cell proliferation and tumors (Aszterbaum et al., 1998; Boukamp, 2005; Göppner and Leverkus, 2011; Scales and de Sauvage, 2009; Sehgal et al., 2014; Xie et al., 1998). Pathogenic mutations in PTCH1 gene have been reported in up to 70% of sporadic human BCCs (Lee et al., 2013). So abrogation of this pathway is considered to be a key cause of BCC development. 

Clinically, BCC is a slowly growing tumor. Several clinical subtypes can be recognized, however, histopathological features are used for definitive identification of BCC subtype (Arits et al., 2011; Raasch et al., 2006). 

Treatment options in patients with BCC are usually made on the basis of an estimate of the risk of recurrence. Patients with high risk BCC subtype have a higher risk of tissue invasion, recurrence, perineural invasion and spread along nerve sheath which can increase local damage (Walling et al., 2004).

In addition, wider spread beyond visible clinical margins increases the risk of BCC recurrence and hence more morbidity due to further tissue damage and the need for more treatment which also adds to morbidity especially when tumor is located near a cosmetically important structure.

In this study we assessed the level of expression of PTCH1 protein in high risk and in low risk BCC subtypes, the outcomes of immunohistochemical analysis of the two groups were examined and compared. 

We found that the BCC predominating in patients older than 60 years (63.4%) and men were more than women with a ratio of 1.66:1. These results are similar to those reported in the majority of studies (McCormack et al., 1997) but differ from other literature in which there was a significant increase of the incidence of BCC in females group and greater incidence of the disease in age distribution between (40- 60) years (Ferreira et al., 2013). In both genders tested in this study, there was a marked predominance of BCC in the face and neck regions (100%). These regions are the main sits of sun exposure and ultraviolet radiation and this is consistent with other studies which show that sun exposure is one of the main risk factors for BCC development (Gailani et al., 1996; Gallagher et al., 1995).

Our data shows that the most prevalent histological subtype was nodular (low risk) which was found in the majority of samples (56/ 101) followed by high-risk BCC subtypes, the infiltrative and micronodular subtypes which were found in (22/101) and (14/101) respectively. Similar to our data, other studies have also shown that nodular BCC is the most prevalent subtype (Jacobs et al., 1982; Ferreira et al., 2013).

The immunohistochemistry results of this study showed varying levels of PTCH1 protein expression in different BCCs subtypes. Positive PTCH1 protein expression was seen in the majority of the examined samples (86/101) while the rest of samples showed negative expression. This data is in agreement with data from the other studies, Undén et al. 1997, found that PTCH1 mRNA was overexpressed in the tumor cells of both familial and sporadic BCC cases (Undén et al., 1997). PTCH1 is involved in down-regulating its own transcription, so deactivating mutations of the *PTCH1 *could possibly lead toPTCH1 mRNA overexpression due to negative feedback loss (Goodrich et al., 1996; Undén et al., 1997). In addition, mouse models studies showed that abrogation of ptch-sonic/hedgehog signaling pathway was crucial for basal cell tumorigenesis process. Kang et al., demonstrated that the overexpression of *Ptch1 in K14PtchFVB* mice model drives skin carcinogenesis and other developmental abnormalities (Kang et al., 2013). 

In this study, the correlation between high risk or low risk BCC subtypes and PTCH1 expression level was not statistically significant (p>0.05), but the data showed that frequency of positive PTCH1expression was higher in low risk BCC subtypes compared to high risk BCC subtypes.

A study by Marcelina et al, indicated that high risk BCC subtypes were more associated with PTCH1 expression. However, in their study only three cases of lower risk subtypes were included hence no firm conclusions can be drawn. Generally speaking the literature is very lacking regarding PTCH1 expression in various BCC subtypes.

In conclusion, PTCH1 expression was detected in majority of BCC cases. However, in this study, no clinical or histological criteria had any significant predictive value for level of PTCH1 expression. More studies especially including higher numbers of various BCC subtypes are needed to examine PTCH1 expression. Furthermore, testing the protein expression level of other components of the Hedgehog (Hh) pathway may predict the aggressiveness of BCC subtypes.
